# The Portuguese Beacon: sharing genomic variant data safely

**DOI:** 10.1093/database/baag012

**Published:** 2026-03-17

**Authors:** Jorge S Oliveira, Sara Sant’Ana, Miguel Santos, Daniel Faria

**Affiliations:** INESC-ID, Instituto Superior Técnico, Universidade de Lisboa, Rua Alves Redol 9, 1000-029 Lisboa, Portugal; BioData.pt, Rua da Quinta Grande 6, 2780-156 Oeiras, Portugal; INESC-ID, Instituto Superior Técnico, Universidade de Lisboa, Rua Alves Redol 9, 1000-029 Lisboa, Portugal; INESC-ID, Instituto Superior Técnico, Universidade de Lisboa, Rua Alves Redol 9, 1000-029 Lisboa, Portugal; BioData.pt, Rua da Quinta Grande 6, 2780-156 Oeiras, Portugal; INESC-ID, Instituto Superior Técnico, Universidade de Lisboa, Rua Alves Redol 9, 1000-029 Lisboa, Portugal

## Abstract

Projects such as the European 1+ Million Genomes initiative and the European Genomic Data Infrastructure project are paving the way towards the age of genomic medicine. To address the challenge of balancing genomic data privacy with biomedical research, the proposed solution is to enable discovery of private datasets through public metadata. Yet enabling data discovery based on genomic variants present in a dataset—which is the goal of Beacon—raises the risk of re-identifying data subjects. We have implemented a Portuguese Beacon endpoint within the scope of the European Genomic Data Infrastructure project, which features a re-identification prevention algorithm to ensure the privacy of data subjects—the first Beacon endpoint to do so. We assessed the impact of the algorithm on data discovery, which varies with the size of the Beacon dataset.

## Introduction

The age of genomic medicine is emerging, enabled by the increasing affordability of DNA sequencing technology, and spearheaded by projects such as the European 1+ Million Genomes initiative [1] and the European Genomic Data Infrastructure (GDI) [2]. This influx of human genomic data stands to benefit healthcare provision by unveiling the genomic causality of diseases—or susceptibility thereto—as well as by enabling the tailoring of treatments to the genomic profile of the patients [1].

However, the road towards realizing this promise is fraught with legal and ethical hurdles due to the sensitivity of human genomic data, which can be personally identifying even in small fragments (e.g., in the case of rare genomic variants), and encode information with ample potential for misuse, including ancestry, sex, family, and susceptibility to diseases [3]. Thus, access to genomic datasets must be restricted and subject to the approval of an ethics committee. Yet, it is imperative that genomic datasets be readily findable, so that researchers with *bona fide* interest can discover them and apply for access.

The European Genome-Phenome Archive (EGA) fulfills this exact purpose as a repository for secure storage of and restricted access to human genomic datasets, where the metadata are publicly available to enable findability [4]. Moreover, it is evolving into a federated solution, with a central node hosting the metadata and acting as a broker to local nodes hosting the genomic data, in order to comply with state laws that require the genomic data of their citizens to be stored within their borders.

Fulfilling the same purpose is Beacon, a data discovery API focusing on genomic variant data, proposed by the Global Alliance for Genomics & Health (GA4GH) [5] and implemented by genomic data repositories worldwide [6]. But whereas EGA enables data discovery from a study/disease perspective, Beacon enables data discovery from a genomic variant perspective, which, even in the simplest case of Boolean queries about individual genomic variants, opens the door to genomic re-identification.

Indeed, Shringarpure and Bustamante [7] showed that it is possible for a user to re-identify a data subject in Beacon given unrestricted Boolean variant queries. It should be noted that, as in all other cases of genomic re-identification, re-identification through Beacon requires prior knowledge of the data subject’s genome (or that of a close relative); although limited knowledge may suffice, particularly if the data subject has rare genomic variants [8]. Moreover, the risk of re-identification is aggravated by access to metadata queries, which were introduced in Beacon v2 and are not contemplated in the aforementioned studies.

The potential consequences of Beacon re-identification are less evident than the risks, as the statistical nature of Beacon queries ensures that the would-be attacker cannot gain knowledge about the genome of their target beyond what they already had. However, ascertaining that a given individual is a member of a given Beacon cohort is a breach of privacy in and of itself, and may enable discovery of phenotypical information about the individual that was not previously known to the attacker, such as disease status, through metadata at the resource, cohort or individual level [7,8]. Thus, to fully comply with the European General Data Protection Regulation (GDPR), a Beacon endpoint in Europe should only allow datasets to be queried by users who already have full access to them, which would essentially render Beacon useless for discovery of private datasets.

This paper presents the Portuguese Beacon, a repository for hosting genomic variant data from Portugal and exposing it through the Beacon v2 API, while ensuring data privacy through the implementation of a Re-Identification Prevention (RIP) algorithm following the budget strategy proposed by Raisaro et al. [8]. Our aim was to enable full integration of the Portuguese EGA and Beacon databases, to maximize the findability of the data incoming from the 1+MG initiative without compromising data privacy.

## Related work

### Beacon

Beacon was proposed at the first meeting of the GA4GH as a simple data discovery API that allowed researchers to find whether a particular genomic data repository contained instances of a genomic variant in which they were interested [5]. Beacon was formally adopted as a GA4GH standard in 2018 [9], and as of 2022 is in release v2 [6].

Beacon v2 defines a number of subtypes of genomic variant queries, as detailed in Table 1, enabling users to search for: the variants with the specified location and **sequence**; all known variants (with optionally specified characteristics) within a specified **range** of the genome; all known variants (again with optionally specified characteristics) occurring within the specified **Gene**; all known variants (optionally with specified type) occurring within a **bracket** of genomic ranges, designed to capture structural variants; the genomic variant corresponding to the specified *genomic allele* (in short form); or the genomic variant corresponding to the specified **amino acid change** in the specified gene.

**Table 1:** Beacon v2 genomic variant query subtypes.

Query Subtype	Parameter	#
Sequence	*referenceName*	1
	*start*	1
	*referenceBases*	1
	*alternateBases*	1
Range	*referenceName*	1
	*start*	1
	*end*	1
	variantType*	0-1
	alternateBases*	0-1
	aminoacidChange*	0-1
	*variantMinLength*	0-1
	*variantMaxLength*	0-1
Gene	*geneId*	1
	variantType*	0-1
	alternateBases*	0-1
	aminoacidChange*	0-1
	*variantMinLength*	0-1
	*variantMaxLength*	0-1
Bracket	*referenceName*	1
	*start*	2
	*end*	2
	*variantType*	0-1
Genomic Allele	*genomicAlleleShortForm*	1
Aminoacid Change	*geneId*	1
	*aminoacidChange*	1

Note: # indicates the cardinality of the parameter, with ‘0-1’ signifying the parameter is optional, and ‘1’ or ‘2’ that it is mandatory (in the respective cardinality). Parameters marked with * are mutually exclusive.

Responses to Beacon v2 queries have three types:

- Boolean – “yes” if any record satisfies the query or “no” otherwise (the only option available in Beacon v1)
- Count–the number of records that satisfy the query
- Record–additional information (typically phenotypical or medical) on the records that satisfy the query

Beacon v2 also contemplates grouping concepts such as *dataset* and *cohort*, enabling queries that target specific data sources within a larger Beacon instance. To define the level of access of each dataset, Beacon v2 employs a three-tiered access model whereby datasets can be:

- Public access–accessible to any user without requiring authentication
- Registered access–accessible to registered users with researcher status
- Controlled access–accessible only to users having explicit authorization (typically granted by a Data Access Committee upon a legitimate access request)

Access levels may be configured independently by response type. For instance, for a given dataset, access to Boolean responses could be Public while access to Count responses is Registered and access to Record responses is Controlled.

Beacon was identified as a key component for data discovery by the ELIXIR Federated Human Data community [10] and more recently by the GDI Project [2], of which Beacon is part of the “Starter Kit” that partners are expected to implement. In both cases, the underlying vision is to establish a network of Beacon endpoints that enables federated data discovery while enabling data providers to maintain control over their own data and uphold data privacy regulations [6].

To support this vision, a reference implementation of Beacon was developed by Rueda et al. [11], so that interested parties without a preexisting genomic variant database can implement a Beacon endpoint from the ground up. This reference implementation consists of four components [11]:

- A genomic variant database implemented in MongoDB
- A set of tools for extraction, transformation and loading of metadata, phenotypic data and genomic variants into the database
- The Beacon v2 query engine implemented as a REST API
- The UK1 synthetic dataset from the CINECA project (https://www.cineca-project.eu/cineca-synthetic-datasets),provided as an example for testing purposes

Alternative reference implementations have been proposed more recently, namely Bycon (https://bycon.progenetix.org/) and EGA-archive Beacon v2 Production Implementation (B2PI) (https://github.com/EGA-archive/beacon2-pi-api), but were not available at the time of our initial development.

### Access control infrastructure

One central aspect in hosting human genomic data, due to its sensitive and personally identifying nature, is a strict access control infrastructure that ensures that users can only view data that is public or to which they have been given explicit access. Functionalities of such an infrastructure must include user authentication, authorization management, and access request management. Moreover, in federated data ecosystems, such as the one being set up by the GDI project, the access control infrastructure must be shared by all nodes of the ecosystem and the aforementioned functionalities should also be federated.

The GDI project has selected three core components for its access control infrastructure, all of which result from efforts to address access control in a federated ecosystem: the Life Science Authentication and Authorization Infrastructure (LS AAI) (https://lifescience-ri.eu/ls-login/), the GA4GH Passport specification[12], and the Resource Entitlement Management System (REMS) (https://www.elixir-finland.org/en/aai-rems-2/).

LS AAI is a centralized service for federated authentication and authorization, developed under the EOSC-Life project and hosted by the European Life Science Research Infrastructures. Authentication in the LS AAI is delegated to the home organization of the user (e.g., a university or research institution) or to one of a few other third-party identity providers (namely Apple, GitHub, Google, LinkedIn and ORCID). Authorization to registered and controlled access data in the LS AAI relies on the Passport specification, an open standard to encode and communicate data access permissions of users through ‘passports’ and ‘visas’, proposed by the GA4GH [12]. Finally, REMS, developed by the Finnish IT Center for Science (CSC), provides access request management functionalities, enabling users to request access to datasets and data controllers to process the requests and manage access rights, which are issued as GA4GH visas.

### Re-identification risks in Beacon

Even considering only Boolean responses on the presence of genomic variants, the lack of query limits in Beacon enables the possibility of re-identifying data subjects [7,8]. Shringarpure and Bustamante [7] demonstrated that re-identification was possible through a statistical test that only relies on the number of “Yes” answers from Beacon for queries about known genomic variants of the target. Raisaro et al. [8] showed that the attack strategy could be optimized if the attacker exploits the population frequency of genomic variants (which can be readily found in public databases) and selectively targets rarer genomic variants first.

Statistical re-identification of individuals through Beacon relies on a likelihood ratio test between the competing hypotheses:

-
$H_0$ : The target’s genome is not in Beacon.
-
$H_1$ : The target’s genome is in Beacon.

The log-likelihood of a series of Boolean Beacon responses $R = \lbrace x_1, \ldots , x_n\rbrace$ is given by [7]:


(1) \begin{eqnarray*}
L(R) = \sum _{i=1}^{n} x_i \log \Pr (x_i = 1) + (1 - x_i) \log \Pr (x_i = 0).
\end{eqnarray*}

And the log-likelihood ratio test is calculated by subtracting the log-likelihood of the series under $H_0$ and $H_1$, which under the optimal attack from Raisaro et al. is given by [8]:


(2) \begin{eqnarray*}
\Lambda = L_{H_0}(R)-{L_{H_1}(R)} = \sum _{i=1}^{n} \log \left( \frac{(1 - f_i)^2}{\delta } \right) \\
+ \log \left( \frac{\delta }{(1 - f_i)^2} \cdot \frac{1 - (1 - f_i)^{2N}}{1 - \delta (1 - f_i)^{2N-2}} \right) x_i
\end{eqnarray*}

where $f_i$ is the frequency of variant *i* in the population, *N* is the number of data subjects in the Beacon cohort queried, and $\delta$ is the probability of sequencing errors and/or variant calling discrepancies (i.e., disagreement between the attacker’s knowledge of the target’s genome and the information in Beacon). However, as Raisaro et al. point out, from a perspective of preventing re-identification, we should consider the best-case scenario for the attacker, which is when their knowledge of the target’s genome perfectly matches the information in Beacon, i.e., $\delta =0$ [8]. Under this scenario, all Beacon queries in the attacker’s series must have a “yes” answer, or the target cannot be in Beacon (i.e., $\Lambda = \infty$). With only “yes” answers possible, equation (2) can be simplified to [8]:


(3) \begin{eqnarray*}
\Lambda = \sum _{i=1}^{n} \log \left( 1 - (1 - f_i)^{2N} \right)
\end{eqnarray*}

Intuitively, the attacker gains more certainty about the presence of the target in Beacon (favoring $H_1$ over $H_0$) the more variants they query with positive results, the rarer those variants are, and the smaller the Beacon cohort is.

Raisaro et al. [8] also proposed three strategies to mitigate the risk of re-identification in Beacon while still enabling its use for data discovery, albeit necessarily limited. However, as the authors demonstrate, the first two of these strategies—excluding variants that are present in only one data subject, and adding noise by answering at random with a given predefined probability—make re-identification harder but do not actually impede it, while affecting the data discovery aspect of Beacon irrespective of the actual risk of the queries. The third strategy, or *budget strategy*, is the only one that effectively impedes re-identification by assigning a log-likelihood budget to each Beacon user for each data subject, which is reduced each time a query from the user encompasses that data subject by an amount corresponding to the statistical power of the query, thereby limiting the statistical power of re-identification attacks to a predefined maximum significance level (set to 0.95 in the authors’ experiments). The only limitations of this strategy are that it requires users to be authenticated and have a single user account, and that it does not factor in the possibility of collusion between users.

## Implementation

### Beacon

The starting point for the Portuguese Beacon was the Beacon v2 reference implementation [11], which was deployed in a virtual machine hosted at the high performance computing infrastructure of the Instituto Superior Técnico. However, the reference implementation includes only the core components required to set up a simple instance of Beacon: a database and an implementation of the Beacon REST API for that database. No access control framework infrastructure is included in the reference implementation, nor indeed is it contemplated in the design of the database and REST API, which lack the capability to filter results by dataset according to access level.

With this in mind, we modified the database and the API to comply with our needs, enabling LS AAI authentication, GA4GH Passport controlled access, and changing the implementation to present results aggregated by dataset. These modifications came with performance drawbacks, requiring the addition of indexes to mitigate them. All the aforementioned changes are documented in detail in Appendix 1.

### Re-identification prevention algorithm

As demonstrated by Shringarpure and Bustamante [7] and discussed in the Related Work section, Beacon enables re-identification of data subjects through unlimited queries targeting their previously known genomic variants. The incorporation of the GDI access control infrastructure in Beacon provides a means to address this risk: non-public datasets added to Beacon should be defined as *controlled access* and only public datasets defined as *public access*, thereby ensuring that users cannot gain any information with Beacon that they could not gain otherwise. However, this perspective also renders Beacon essentially useless for data discovery: if users can only see datasets to which they already have gained explicit access, they can never find new datasets of interest through Beacon to request access.

To address this limitation, we wanted to allow limited access to restricted or controlled datasets to an extent that enables data discovery but renders re-identification impossible. This is possible by implementing the *budget strategy* proposed by Raisaro et al. [8] to impede re-identification attacks, which restricts the amount of statistical power a user can gain about any data subject. As the goal is to use this strategy to enable data discovery, it should apply only to datasets that (authenticated) users do not have access, as detailed in Table 2, and should enable them to perform only simple genomic variant queries over those datasets. Moreover, queries under this strategy should yield only Boolean results, as the statistical test is based on this assumption. Record results would not only provide additional information facilitating re-identification, but also aggravate the potential consequences, so they clearly should not be allowed under this scenario. Using Count results would be a possible alternative, but would require updating the statistical test to consider the Beacon frequency explicitly. Since this would likely lead to budgets running out more rapidly, and given that Boolean results are sufficient for data discovery, we opted for the latter in implementing the *budget strategy* in our Beacon, labeling it the Re-Identification Prevention (RIP) algorithm.

**Table 2:** Applicability of the Re-Identification Prevention (RIP) algorithm.

User status	Dataset access level		
	Public	Registered	Controlled
Unauthenticated	Full access	No access	No access
Authenticated	Full access	RIP	RIP
Researcher	Full access	Full access	RIP
Authorized researcher	Full access	Full access	Full access

As it is a critical aspect of our implementation, we reiterate that the RIP algorithm is only applied in cases where an authenticated user does not have explicit access to a registered or controlled dataset, so as to enable the use of Beacon for data discovery. Public datasets or those to which users have explicit access are unaffected by the RIP algorithm. Thus, in our implementation the RIP algorithm does not in any way restrict the use of Beacon, but rather extends it, giving (very limited) access in cases where there would not be any. As the algorithm ensures a user can never gain statistically significant information to re-identify any data subject, data accessed under the algorithm can be considered anonymised and thus safe to share under the GDPR.

Implementing the RIP algorithm required adding allele frequency data to the Beacon database. We retrieved this data using the Ensembl REST API (https://rest.ensembl.org) and updated each variant document in the database accordingly. It also required adding two additional data objects to the Beacon database, *history* and *budget*, to track respectively the query history of each user and the budget they have for each data subject—the key component of the RIP algorithm. The *history* object records the user ID, the dataset ID, the query they submitted under the RIP algorithm, and the respective response returned by the RIP algorithm, so as to enable repeated queries from the same user to be recalled without affecting their budget. The *budget* object records the user ID, the IDs of all data subjects encompassed by their past queries, and the remaining budget they have for each data subject, which translates to how much statistical power they can still safely gain on the data subject (i.e., without risking re-identification).

**Figure:**
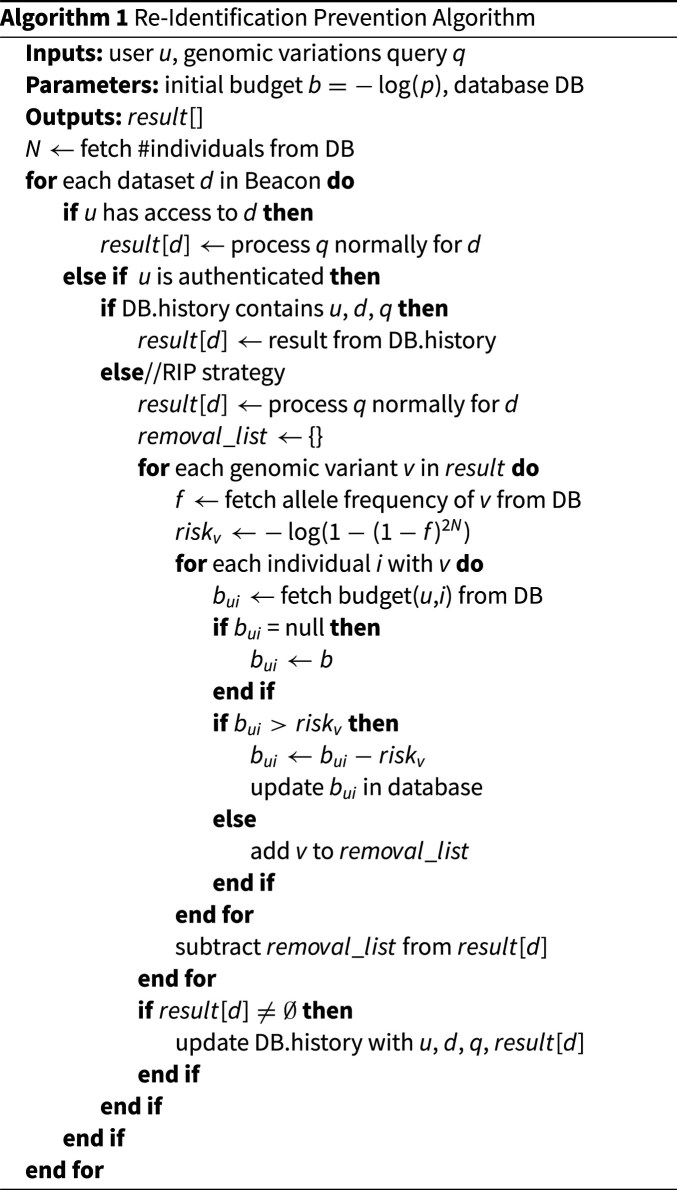


The RIP algorithm is detailed in Algorithm 1. For each dataset to which the algorithm applies, if the user has previously made the query, the stored result they obtained previously is returned, and no changes are made to their budget. Otherwise, for each genomic variant returned by the query, the re-identification risk the query poses to each data subject with the genomic variant is calculated, and if the risk exceeds the budget of the user for any such data subject, the variant is removed from the results.

The initial budget set for each {user, data subject} pair is given by $-\log (p)$, where *p* is the lowest p-value we want to allow a user to reach when attempting to re-identify the data subject based on their genomic variants. The value of *p* should evidently be higher than the highest p-value normally considered significant in statistical testing, i.e., 0.1, in order to effectively preclude re-identification by any individual attacker. However, the risk of re-identification by colluding users still exists, and because of that risk, we cannot publish the exact value of *p* in use in our Beacon, as it would enable colluding users to optimize their strategy, as we will elaborate in the Evaluation section.

## Evaluation and discussion

### Performance assessment

As the execution of the RIP algorithm encompasses multiple additional queries to the database, we assessed its impact on the performance of Beacon by running 100 distinct genomic variant queries with and without the RIP algorithm. Since the queries were performed over the CINECA UK1 synthetic dataset, its access was set to *registered* for the tests with the RIP algorithm, so that the algorithm was always applied. The queries were executed on Beacon’s development server, an x86_64 virtual machine with 4 Intel(R) Xeon(R) Silver 4214 VCPUs 2.20 GHz and 8GB RAM, so the run times are not directly comparable with those of the previous assessment. As shown in Table 3, the execution of queries under the RIP algorithm was slower than their normal execution by an order of magnitude. Nevertheless, this increment in execution time is not sufficient to compromise the usability of Beacon, as the absolute time is still under 1 second per query, especially considering that the RIP algorithm will be active only in scenarios where Beacon is to be used for data discovery, where we actually want to prevent users from performing large numbers of queries in a row.

**Table 3:** Performance impact of the RIP algorithm on Beacon.

Mode	Mean run time (ms)
Normal	43
RIP algorithm	553

Note: Performance was assessed by executing 100 distinct genomic variant queries over the CINECA UK1 synthetic dataset, with and without the RIP algorithm.

Given that the implementation of the RIP algorithm also requires additions to the database, its cost in terms of storage must also be assessed. The *budget* database object scales linearly with the number of data subjects in the Beacon database, taking up at most 12 bytes per data subject per user (4 bytes for the id of the subject and 8 bytes for the budget itself) plus 4 bytes per user (for the user id). Thus, in a large Beacon, with 100,000 data subjects, the *budget* object would take at most 1 MB per user. If the Beacon is also popular and has 10,000 users, then the total storage cost of the *budget* would be at most 10 GB, which is certainly not negligible but hardly concerning either. And it should be noted that the expected cost is much lower than the worst case scenario, as the RIP algorithm is only applied to datasets to which the user does not have explicit access, and the budget is only computed and stored in those cases. The *history* object is, in theory, potentially more problematic, as it scales linearly with the number of queries made by each user, which is unbounded and will accumulate over time. However, the *history* is also only stored for datasets to which the user doesn’t have explicit access, with the expected use being a few queries for data discovery. Thus, a large *history* is only likely for a user if they are indeed undertaking a re-identification attempt, and monitoring the size of the *history* can be seen an additional security measure.

### Privacy assessment

To validate that the RIP algorithm was functioning as expected, we conducted simulated re-identification attacks following Raisaro et al.’s “optimal” attack strategy [8]. For 100 different data subjects selected at random from the CINECA UK1 synthetic dataset, a would-be attacker performed targeted Beacon queries about the genomic variants of the subject in ascending order of population frequency (i.e., starting with the rarest) until a query surpassed the remaining budget of the attacker for the target (triggering a false negative response). For each target data subject, we counted the number of successful queries of the attacker until their budget for the subject was surpassed. For the purpose of this testing, we set $p = 0.1$ for the initial budget, which corresponds to the upper limit of statistical significance for re-identification.

**Figure 1:**
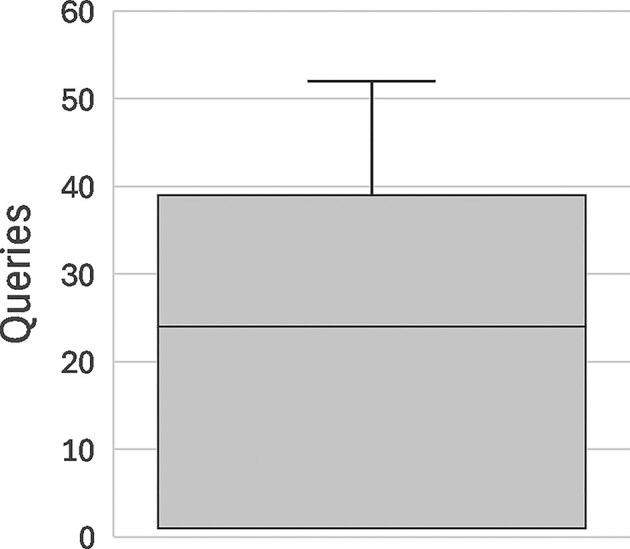
Mean number of queries in data discovery experiments as function of the dataset size, with linear regression line. Experiments consisted of 100 runs of up to 100 random genomic variants for different dataset sizes, until the first data subject is removed from the results by the RIP algorithm.

The results of this assessment are displayed in Fig. 1. Simulated attackers were able to perform an average of 20 queries before their budget ran out and the target of the attack was occluded from the results, with individual attack attempts ranging from 1 to 52 queries until occlusion of the target. These results illustrate how easy it is for an attacker to accomplish re-identification through Beacon—particularly when the Beacon dataset is relatively small—which justifies the need for a measure like the RIP algorithm.

With $p \gg 0.1$ for the initial budget, the RIP algorithm ensures statistically significant re-identification is not possible for an individual attacker, not even if they are aware of the RIP algorithm. However, statistically significant re-identification is possible through collusion between multiple attackers if they are aware of the RIP algorithm (if they are not, they would treat negative responses as true negatives and conclude that their target is not in Beacon). It should be mentioned that the risk of collusion is mitigated to some extent by our Beacon’s use of the LS AAI for authentication, with unauthenticated users unable to query non-public datasets altogether. Nevertheless, the risk exists and must be addressed, or we cannot claim that the RIP algorithm safeguards data privacy.

The reason we do not disclose the exact value of *p* implemented in our Beacon is precisely to foil collusion attacks, as the success of such attacks opposing the RIP algorithm hinges on whether the attackers know the value of *p*. Concretely, a group of savvy colluding attackers that knew *p* could optimize their colluding attack within the constraints imposed by the RIP algorithm, namely by skipping genomic variants of the target subject that are rare enough to surpass *p*, and by tracking their budget and querying about less rare genomic variants in such a way as to maximize it, passing the task to the next attacker once their budget is exhausted. Thus, knowing *p*, a group of users could operate in such a way as to never get a negative response from Beacon unless their target is actually not in Beacon, and effectively escape the confines of the RIP algorithm, reaching any significance level they desire, limited only by the number of attackers and the known genomic variants of the target. Evidently, the higher the value of *p*, the more colluding users are needed to accomplish re-identification, and the more known variants whose rarity is below the threshold of *p*. But even with a value of *p* as high as 0.5, re-identification can be accomplished by only five colluding users if they know the value of *p*.

Without exact knowledge of *p*, the scenario is very different, as colluding attackers cannot avoid getting negative responses, and are forced to consider that these may be either due to the RIP algorithm or due to their target actually not being in Beacon (which *a priori* is the more probable scenario). Thus, their re-identification attack can no longer be modeled by Equation (3), corresponding instead to:


(4) \begin{eqnarray*}
\Lambda = \sum _{i=1}^{n} \log \left( \frac{(1 - f_i)^2}{\rho } \right) \left(1 - x_i \right) \\
+ \log \left( 1 - (1 - f_i)^{2N} \right) x_i
\end{eqnarray*}

where $\rho$ is the probability of a RIP false positive, which equates to the probability of the target being in Beacon, which in turn can be estimated from the previous iteration of $\Lambda$. In general, for a minimally informative genomic variant ($f_i \le 0.01$) and a plausible value of *p*, a negative answer will increase $\Lambda$ in favor of $H_0$, setting-back the re-identification attack—a set-back that will be greater the higher the value of *p*. Empirical evidence has shown that from a certain threshold of *p*, the set-back is sufficient to offset any statistical power gained by an individual attacker up to the point their budget runs out, meaning the following attacker starts at $\Lambda = 0$ and no progress can be made through collusion. By setting a value of *p* within this range, we can make collusion attacks highly unlikely to succeed and so demanding in terms of number of attackers required as to deter any attempt.

### Data discovery assessment

To assess whether the RIP algorithm enables Beacon to still be useful for data discovery with our chosen value of *p*, we simulated researchers conducting queries on their genomic variants of interest until the results were adulterated by the RIP algorithm. For this purpose, we carried out 100 runs (simulating 100 researchers) where Beacon was queried for up to 100 randomly chosen genetic variants in succession, counting the number of runs until the RIP algorithm occluded the first data subject from the results. Since the size of the Beacon dataset strongly affects the impact of the RIP algorithm on data discovery (corresponding to the parameter *N* in the algorithm) we repeated the experiment with different (simulated) dataset sizes: 2504 (the actual size of our test dataset), 10000, 25000 and 100000.

The results of these experiments are summarized in Table 4. They show that, for the actual size of the CINECA UK1 synthetic dataset, data discovery is significantly restricted, with researchers able to obtain unadulterated results for only 1.7 queries on average, and 30% of them getting a false negative on their first query, thus stopping their run at zero successful queries (a “zero run”). However, the prospects improve substantially with even a relatively small increase in dataset size: with a dataset of 10000 data subjects, researchers are able to perform 5 queries with unadulterated results on average, and the number of zero runs drops to 10%. Considering that the scenario being contemplated is that of a researcher looking for datasets that have genomic variants they are interested in so that they can apply for full access, 5 queries seems a reasonable number.

**Table 4:** Data discovery experiment results.

Dataset size (N)	Mean queries	Zero runs (%)
2504	1.7	30
10000	5.0	10
25000	8.3	2
100000	32.5	0

Note: Mean number of queries and number of runs with zero queries in 100 runs of up to 100 random genomic variants until the first data subject is removed from the results by the RIP algorithm, considering different (simulated) dataset sizes.

**Figure 2:**
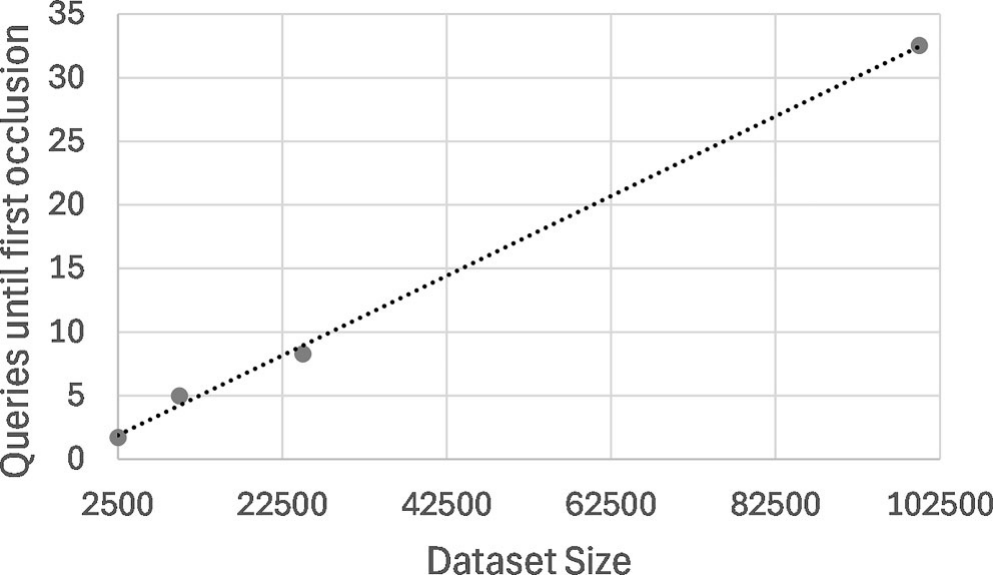
Box-plot depicting the distribution of number of queries until occlusion of the target data subject by the RIP algorithm ($p = 0.1$) in 100 re-identification attacks.

As shown in Fig. 2, the mean number of queries until first occlusion of a data subject varies approximately linearly with the size of the Beacon dataset. With 100000 data subjects, no zero runs occurred, and one researcher was able to perform all 100 queries, with a mean of 32.5 queries that is likely sufficient to go beyond data discovery and enable simple data analysis.

Zero runs occur when the researcher asks first for a rare variant that has a frequency below the minimum frequency allowed by the RIP algorithm for a dataset of that size. For any given dataset size and any initial budget, there may exist variants that are sufficiently rare to exceed the budget. Confirming the expectation, our results indicate that for smaller dataset sizes, even moderately rare mutations can exceed the budget, whereas for larger dataset sizes ($\ge$25000 data subjects), only the rarest mutations do so. Thus, the RIP algorithm does prevent the discovery of small datasets based on a search for rare mutations, but for large enough datasets, this will no longer be an issue.

We note again that the RIP algorithm only applies to datasets to which users do not have explicit access, namely: registered datasets by non-researchers and controlled datasets by both non-researchers and non-authorized researchers. Without the RIP algorithm in place, users would not be able to use Beacon to discover such datasets, whereas with the algorithm they will be able to discover and request access to some of them, even if there may be limitations on the rarity of the variants they are able to discover if the Beacon dataset is small. The RIP algorithm does not affect the normal use of Beacon, in scenarios where the user has explicit access to the dataset.

### Dataset & budget updates

As the calculation of the budget allocated by the RIP algorithm to a user and data subject is based on the size of the dataset, it stands to reason that it should be updated should the dataset grow in size in the future (regardless of whether the budget was exhausted). While the issue has not arisen in our Beacon, we anticipate executing an offline batch update of all budgets every time a dataset increases in size. Since we store the history of all queries made by each user under the RIP algorithm, we can re-compute the budgets from the history.

## Conclusions

Balancing genomic data privacy with biomedical research is one of the greatest challenges we must address if the age of genomic medicine is to ever fully materialize. Enabling discovery of private genomic datasets by publishing enough of their metadata to enable findability is currently seen as the most viable solution. Yet not all metadata is created equal with respect to data privacy: metadata about the context of a study—as available in the EGA—is generally safe to share openly, but metadata about the genomic variants present in the study—as available in Beacon—opens the door to re-identification of the data subjects [7,8].

In our implementation of a Beacon endpoint for Portugal, within the scope of the GDI project, we sought to address the re-identification risk posed by Beacon by incorporating the RIP algorithm, based on the budget strategy proposed by Raisaro et al. [8]. Our aim is to enable our national federated EGA node and Beacon endpoint to be fully integrated and used in concert for data discovery, for which it is essential that the privacy ensured by the two systems is equivalent.

The RIP algorithm is guaranteed to impede re-identification attacks from a single user, and even colluding users are unlikely to succeed if the value of the significance level *p* is kept high enough and not disclosed. However, a high value of *p* also impacts the usefulness of Beacon for data discovery, which is admittedly limited for datasets of relatively small size ($\le$ 2500 data subjects). In particular, the RIP algorithm prevents small datasets from ever being discovered based on queries for rare genomic variants.

The outlook is considerably better for large datasets, and at 100000 data subjects the impact of the RIP algorithm on data discovery becomes mostly negligible. And given the scale of projects such as the European 1+ Million Genomes (1+MG) initiative, a genomic dataset with 100000 data subjects is no longer outlandish, and may soon be a widespread reality. Additionally, to improve the data discovery prospects, small datasets can be pooled together, at least from the point of view of researchers without full access, by only allowing them to query Beacon globally.

As for our Beacon database, we have already incorporated a Portuguese genomic dataset from a colorectal cancer study fully detailed in Golkaram et al. [13], and we expect that, with the addition of the RIP algorithm ensuring the privacy of the data subjects, this will be the first of many datasets.

## Supplementary Material

baag012_Supplemental_Files

## Appendix 1

### Implementation details

To address the missing features in the Beacon Reference Implementation mentioned in Section ‘Implementation’, we had to modify both the database and the REST API. The former was modified by adding a single nonconflicting field, *datasetId*, to every data object under the umbrella of a dataset (e.g. individual, biosample) to enable querying the database by dataset. The latter was modified by changing the scope of all queries from the database level to the dataset level, meaning every Beacon API call is implemented as N identical queries to the database, one per dataset to which the user has access.

With these modifications in place, we integrated the GDI access control infrastructure with our Beacon instance, by incorporating LS AAI for authentication, adopting GA4GH Passports for authorization (which are retrieved from LS AAI and/or a REMS instance), and connecting to REMS for access request management. Thus, unauthenticated users or authenticated users without the researcher status are only able to access data from *public access* datasets, whereas as authenticated users with the researcher status according to their GA4GH Passports can access also *registered access* datasets as well as any *controlled access* datasets granted by the access permissions of their GA4GH Passports.

Alas, these modifications to Beacon calls had a negative impact on the performance of the API, as they multiplied the number of queries made per call. To mitigate this impact, we assessed the effectiveness of the database indexes included in the Beacon reference implementation. These consisted exclusively of composite text indexes over all the fields of each data object (one index per MongoDB collection), designed to optimize text searches across all fields, but immaterial for most Beacon calls. As genomic variants represent the bulk of the Beacon data and are the central objects of the main Beacon calls, we concluded that an index over the *genomicVariations* data object, tailored to optimize queries for both genomic variations and genomic regions, would be advantageous. Thus, we added a new composite b-tree index over the following key fields of *genomicVariations: _info.datasetId, _position.refSeqId, _position.start, variation.referenceBases, variation.alternateBases*.

### Index optimization results

We assessed the effectiveness of the index we added to our Beacon database by executing three distinct genomic variant queries and three distinct genomic region queries with and without the index, on the CINECA UK1 synthetic dataset. Each query was executed six times in the Beacon’s production server, an x86_64 virtual machine with 8 Intel(R) Xeon(R) Silver 4214 VCPUs 2.20 GHz and 16GB RAM. As shown in Table A1, the index had a dramatic impact in the performance of genomic variant queries, speeding them up by three orders of magnitude, and a smaller but still significant impact in genomic region queries, speeding them up by a factor of  5.

**Table A1:** Performance impact of the index added to the Beacon database.

Query type	Mean run time original (ms)	Mean run time with index (ms)
Genomic variant	1587	0.3
Genomic region	1617	293

Note: Performance was assessed in three genomic variant queries and three genomic region queries executed six times each on the CINECA UK1 synthetic dataset, in the virtual machine hosting the Beacon.
